# Theory-based habit modeling for enhancing behavior prediction in behavior change support systems

**DOI:** 10.1007/s11257-022-09326-x

**Published:** 2022-05-31

**Authors:** Chao Zhang, Joaquin Vanschoren, Arlette van Wissen, Daniël Lakens, Boris de Ruyter, Wijnand A. IJsselsteijn

**Affiliations:** 1 Human-Technology Interaction Group, Department of Industrial Engineering and Innovation Sciences, 513, 5600MB Eindhoven, The Netherlands; 2 Digital Engagement, Cognition and Behavior Group, Philips Research, High Tech Campus 34, 5656AE Eindhoven, The Netherlands

**Keywords:** Habit formation, Dental behavior change, Computational models, Predictive modeling, Digital health intervention

## Abstract

Psychological theories of habit posit that when a strong habit is formed through behavioral repetition, it can trigger behavior automatically in the same environment. Given the reciprocal relationship between habit and behavior, changing lifestyle behaviors is largely a task of breaking old habits and creating new and healthy ones. Thus, representing users’ habit strengths can be very useful for behavior change support systems, for example, to predict behavior or to decide when an intervention reaches its intended effect. However, habit strength is not directly observable and existing self-report measures are taxing for users. In this paper, building on recent computational models of habit formation, we propose a method to enable intelligent systems to compute habit strength based on observable behavior. The hypothesized advantage of using computed habit strength for behavior prediction was tested using data from two intervention studies on dental behavior change ($N = 36$ and $N = 75$), where we instructed participants to brush their teeth twice a day for three weeks and monitored their behaviors using accelerometers. The results showed that for the task of predicting future brushing behavior, the theory-based model that computed habit strength achieved an accuracy of 68.6% (Study 1) and 76.1% (Study 2), which outperformed the model that relied on self-reported behavioral determinants but showed no advantage over models that relied on past behavior. We discuss the implications of our results for research on behavior change support systems and habit formation.

## Introduction

Behavior change support systems (BCSSs) are digital systems that support users to change their behaviors in desirable ways such as living a healthier or more sustainable lifestyle (Oinas-Kukkonen 2013; Lathia et al. 2013). To facilitate behavior change, BCSSs may use the means of education, persuasion (Fogg 2002; IJsselsteijn et al. 2006), or a combination of theory-based behavior change techniques (Abraham and Michie 2008). In many application domains where behaviors are repeated frequently, such as when promoting healthy lifestyles, one of the challenges for successful change is the task of breaking bad old habits and forming healthy new habits (Gardner and Rebar 2019; Karppinen et al. 2018; Pinder et al. 2018). Habitual behaviors are characterized as automatic responses triggered by cues in the environment (e.g., eating crisps when watching TV) or by goals activated in one’s working memory (e.g., using a bike when commuting to work) (Sheeran et al. 2005; Wood and Neal 2007). The lack of deliberations of behavioral consequences explains why bad habits persist even when they conflict with one’s current goals (Dickinson 1985). On the bright side, when a good habit is formed, it helps behavioral maintenance and prevents relapses. Modeling users’ habits can potentially increase the effectiveness of BCSSs.

Although the term “habit” is intuitively understood by most people, it is important to clarify what we mean by “habit” in this paper. In the field of ubiquitous computing, modeling habits usually refers to the modeling of users’ actual behaviors, i.e., detecting and recognizing recurrent behavioral patterns and routines (Kalantarian et al. 2015; Meng et al. 2017; Shoaib et al. 2015), sometimes contingent on specific user contexts (Banovic et al. 2016). In contrast, based on psychological theories (Marien et al. 2019; Sheeran et al. 2005; Verplanken et al. 2018; Wood and Neal 2007; Wood and Rünger 2016), we define habits as the cognitive associations between user behaviors and the triggering user contexts, thus separating habits from habitual behaviors themselves. The strengths of these associations (or simply habit strengths) build up through context-dependent behavior repetitions and they in turn increase the probability that the behavior is performed in the same context.

Modeling the habit strength of a particular user behavior can benefit BCSSs in at least two ways. First, assuming a causal effect of habit on behavior, knowing the habit strength can assist a system to predict a user’s behavior more accurately. Accurate behavior prediction is the basis for personalizing interventions, for example, sending a reminder when the system predicts that the user is unlikely to perform the desirable behavior on their own. Second, it is widely acknowledged that reminders in many so-called “habit-formation” apps induce behavior repetition but hinder the formation of real habits that are supposed to be connected to environmental cues (Renfree et al. 2016; Stawarz et al. 2014, 2015). Thus, representing habit strength as a cognitive state enables a system to distinguish genuine context-driven habitual behaviors from repeated behaviors that are simply prompted by digital systems. It also allows a system to decide when to withdraw proactive interventions on a specific behavior, knowing from the model that the user’s behavior will likely be maintained by the strong habit alone.

Habit strength can be measured using the Self-report Habit Index (SRHI) (Verplanken and Orbell 2003) or its behavioral automaticity sub-scale (Gardner et al. 2012). Although these questionnaires can be implemented in a BCSS on a daily basis, they pose a burden to users and may suffer from memory and social desirability biases and even interfere with primary intervention techniques. Recently developed theory-based computational models of habit formation provide a new approach of quantifying habit strength based on observable behavior and context (Klein et al. 2011; Miller et al. 2019; Psarra 2016; Tobias 2009), but the usefulness of these models has not been extensively tested in real-world behavior change interventions. In this paper, we test whether computing habit strength and related variables based on existing computational models improves behavior prediction in two real-world intervention studies on dental behavior change. If the theory-based approach outperforms theory-free predictive models in behavior prediction, the results provide empirical support for the more widespread use of computational models and encourage other use cases of computing habit strength, such as intervention personalization.

In the remainder of the paper, we start with the theoretical background of our work, followed by the overall modeling and evaluation approach. Next, the data-collection method and results of the two field studies are presented. The paper concludes with a general discussion, including implications for designing more personalized BCSSs.

## Theoretical background

### The psychology of habit

Habits are formed through behavior repetitions in the context of goal-directed learning (Marien et al. 2019; Wood and Neal 2007; Wood and Rünger 2016). According to the fundamental principles of reinforcement learning in humans and animals (Postman 1947; Sutton and Barto 2018; Thorndike 1932), given a goal and a context (e.g., search for food in a cage), a learner learns the associations between their behaviors and outcomes through trial and error and the behavior with the highest probability of obtaining positive outcomes is repeated more and more frequently (e.g., a rodent pressing a lever to obtain pellet). Crucially, in addition to this response-outcome learning (or goal-directed learning), the learner also picks up an association between the context (or stimulus) and the behavior, referred to as stimulus-response learning or habit learning. Decades of psychological research has shown that goal-directed learning and habit learning play distinct roles in animal and human behavioral control (Dickinson 1985; Thorndike 1932) and the two types of learning have distinct underlying brain regions (Yin and Knowlton 2006). This effect of context-specific behavior repetition on habit strength provides the basis for habit modeling.

Two addition points regarding habit formation are worth noting. First, in this cognitive perspective, habits and habitual behaviors are context-specific. Even when the behavior seems to be the same at the level of motor control, performing the same behavior in two different contexts should be considered as two different habits, with potentially different habit strengths. For example, one person may have a strong habit of brushing their teeth after getting up in the morning, but only starts to form a new habit of brushing their teeth before sleep in the evening. Second, while the initial behavior repetitions can be driven by different motivational factors (e.g., intrinsic enjoyment of the behavior, external reward, or even coercion), it is generally assumed that the accompanied habit learning (the build-up of a context-behavior association) follows exactly the same principle. Therefore, a general habit modeling approach is theoretically applicable to a wide range of behaviors.

Once a strong habit is formed, habit strength as a cognitive construct reinforces the associated behavior. When the same context is encountered or the same goal is activated, this association immediately brings a representation of the behavior into one’s working memory (Tobias 2009) or enhances the baseline preference signal of the behavior in decision-making (Roe et al. 2001; Zhang 2019). Both these mechanisms increase the probability that a behavior is repeated in the same context. This reciprocal effect of habit strength on actual behavior provides the rationale for using computed habit strength for behavior prediction.

### Computational models of habit learning

Following the theories of habit, Klein and colleagues (2011) proposed a computational model that formally accounts for the relationship between behavior repetition and habit strength. The basic idea of the model was inspired by the Hebbian learning principle in neuroscience (Hebb 1949): assuming a network of cognitive nodes representing behaviors and contextual cues, the link between a *behavior* node and a *cue* node is strengthened whenever the two nodes are activated at the same time, i.e., when the behavior is performed with the presence of that particular cue in the environment.

**Fig. 1:**
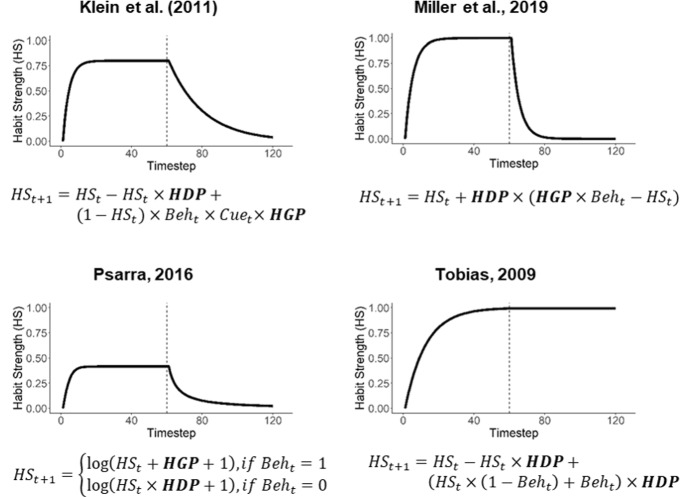
Equations of different computational models of habit learning and their simulation results under a simple scenario where the target behavior is consistently performed from step 1 to 60 but is abandoned from step 61 to 120 (the vertical dotted line separates the two phases). (HS, habit strength; Beh, behavior; HDP, habit decay parameter; HGP, habit gain parameter. Note that we unified the original parameter names for the clarity of presentation, but their exact meanings are bounded by each of the equations)

Figure 1a shows the mathematical equation of the model and a simulation result of how habit strength changes over time in a simple scenario and with plausible values for the model parameters. When a behavior is consistently performed in the first half of the simulation, habit strength increases over time but the rate of growth decreases so that habit strength approaches a plateau. When the behavior is abandoned in the second half, habit strength decays proportionally but at lower rate than the habit growth. These basic patterns are consistent with the empirical data from a field study on habit formation where participants reported their habit strength using the SRHI (Lally et al. 2010). In addition to the model by Klein and colleagues, other very similar models of habit formation have been developed over the years and in various application contexts (Miller et al. 2019; Psarra 2016; Tobias 2009). Figure 1b–d illustrate the very similar model simulation results^1^. For our purpose of testing the usefulness of the general modeling approach for behavior prediction, we decided that it was sufficient to focus on the Klein et al. (2011)’s model^2^.

### Habit formation and memory process

Habit formation is also closely related to memory processes. In order to perform a desirable behavior (e.g., brushing one’s teeth before going to sleep), a person first needs to recall the behavioral option before they evaluate it with competing options (e.g., going to bed directly) (Kamphorst and Kalis 2015; Zhang et al. 2021). A similar idea was presented in B.J. Fogg’s behavior model for persuasive design that even with sufficient motivation and ability to perform a behavior, a trigger for the behavior is often needed (Fogg 2009). When a strong habit is formed, the context in which a behavior has been repeatedly performed can function as such a trigger by itself (Psarra 2016; Tobias 2009; Wood and Neal 2007). In contrast, when a habit is still weak, a newly learned behavior can be “forgotten” in relevant contexts and this requires additional triggers such as reminders from a BCSS.

In addition to modeling habit formation, Tobias (2009) also proposed a computational model of how memory accessibility of behavioral options changes over time. Like any other memory process, the accessibility of a behavioral option decays gradually over time but can be restored upon receiving reminders or when the behavior is performed. Other unobservable factors, such as the mental rehearsal of an option (Einstein and McDaniel 1996), also influence accessibility but their effects are integrated into a single decay parameter in Tobias (2009). The equation is formally introduced in the next section.

## Modeling and evaluation approach

### Computing habit strength and memory accessibility

Based on the theories and computational models reviewed, we focus on two cognitive quantities that can be computed by a digital system. Of our primary interest, the habit strength of a target behavior for a user in a behavior change process is computed based on Klein et al. (2011)’s model. The equation with a habit decay parameter (HDP) and a habit gain parameter (HGP) is as follows:

1 $$\begin{aligned} \text {HS}_{t+1} = \text {HS}_{t} - \text {HS}_{t} \times {\text {HDP}} + (1 - \text {HS}_{t}) \times {Beh}_{t} \times {Cue}_{t} \times {HGP} \end{aligned}$$

The equation implies that given an initial habit strength of a user (HS$_0$), the subsequent habit strength at any time point (HS$_t$) can be computed as long as the past occurrences of behavior (Beh) and cues (Cue) are known. In an empirical study or a behavior change application, users can be asked to self-report their habit strengths at the beginning and the self-reported values (scaled to [0, 1]) can be used as initial values. Both actual behavior and environmental cues can be potentially monitored by sensors in a BCSS. In the current research, we make a simplifying assumption that users always perform the target behavior in the same context (i.e., participants in our studies always brushed teeth in their own bathrooms and at similar time), so the variable Cue$_t$ is always 1.

In addition to habit strength, the memory accessibility of a behavioral option can be computed using the equation in Tobias (2009). Accessibility (Acc) decays naturally as a natural memory process, but can be enhanced by behavior executions (Beh) and external reminders (Rem). The equation controlled by three free parameters—accessibility decay parameter (ADP), accessibility gain parameter with behavior execution (AGP$_\mathrm{beh}$), and accessibility gain parameter with reminder (AGP$_\mathrm{rem}$), is as follows:

2 $$\begin{aligned}&\text {Acc}_{t+1} = \text {Acc}_{t} - \text {Acc}_{t} \times {\text {ADP}} + (1 - \text {Acc}_{t})\nonumber \\&\times (\text {Beh}_{t} \times {\text {AGP}_\mathrm{beh}} + \text {Rem}_{t} \times {\text {AGP}_\mathrm{rem}}) \end{aligned}$$

When a user is persuaded by a BCSS to learn a new behavior, the initial value of memory accessibility (Acc$_0$) of the target behavior can be assumed to be 1 (maximum). Subsequent memory accessibility can be easily updated by monitoring actual behavior and reminders sent by the digital system itself. For simplification, any procedure used in our empirical studies (e.g., face-to-face meeting, email communication, etc.) that reminded participants of the target behavior was assumed to restore memory accessibility by the same amount controlled by a single parameter AGP$_\mathrm{rem}$.

### Using computed variables in predictive modeling

**Fig. 2:**
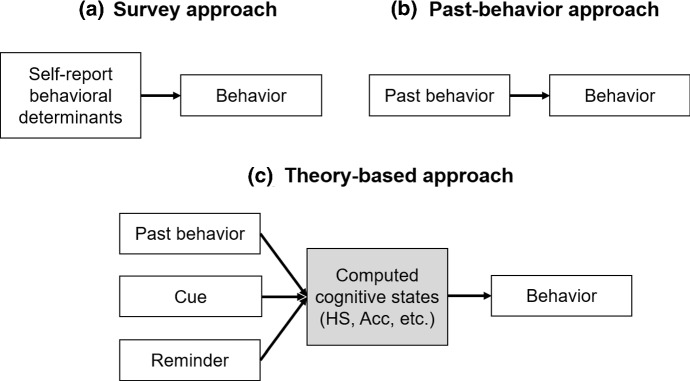
Visual representations of the three different modeling approaches: **a** survey approach; **b** past-behavior approach; **c** theory-based approach

The primary goal of the current research is to evaluate the usefulness of computing habit strength and memory accessibility in the use case of behavior prediction. In a behavior change intervention, predicting future behavior based on information already collected is an important but challenging task. For example, when a user is prompted by a BCSS to brush their teeth every morning, it is a useful task to predict whether they will brush their teeth the next morning (also known as a 1-step forecast) based on all the system knows about the user at that point. A conventional approach for behavior prediction in psychology relies on self-reported behavioral determinants measured by periodical surveys (survey approach, see Fig. 2a), such as attitude, intention, and self-report habit strength (Verplanken and Orbell 2003). Another approach is simply to use past behavior to predict future behavior, for example, by calculating the percentage of days in the past when the user brushed teeth in the morning (past-behavior approach, see Fig. 2b). Instead of these two approaches, the system can also compute habit strength and memory accessibility based on historical data (past behavior, cue, reminder, etc.) and use the computed theoretical quantities to predict future behavior (theory-based approach, see Fig. 2c). Computing the theoretical quantities is fully justified if the *theory-based approach* predicts future behavior more accurately than the *past-behavior approach* and at least as accurately as the *survey approach*, given that it bypasses the need to burden users with questions. Note that we focus on comparing the relative performance of the models rather than optimizing absolute performance.

### Intervention studies for evaluating the models

We used data sets from two intervention studies on dental health behavior to compare the three approaches above. In these two studies, participants were instructed to brush their teeth twice a day for about three weeks, while their brushing behaviors were continuously monitored by sensors, and their attitudes toward toothbrushing and self-reported habit strengths were measured once a week. In the context of dental health, toothbrushing twice a day is recommended by most dentists as an effective way to prevent dental plaque, but research has shown that compliance with this optimal dental routine is not universal (Aunger 2007). For someone who only brushes teeth once a day, for example in the morning, brushing for a second time in the evening would require forming a new habit. This behavior change requires changes in one’s attitude and intention in the beginning, preferably supported by external reminders (e.g., from a BCSS), but with enough practice the behavior should become a habit or part of one’s bedtime routine (Aunger 2007). We chose to study toothbrushing behavior because of its relative simplicity, context stability (e.g., usually in the bathroom at home) and high occurrence frequency, but the general approach of modeling habit should apply to other lifestyle behaviors (Zhang et al. 2021).

Despite the differences between the two studies in their study samples, intervention techniques used, and procedures, for our modeling purpose, they can be considered as two conceptual replications and together they provide a stronger test of our modeling approach. Therefore, we report the methods and results of the two studies in parallel^3^.

## Method

### Design and procedure

**Fig. 3:**
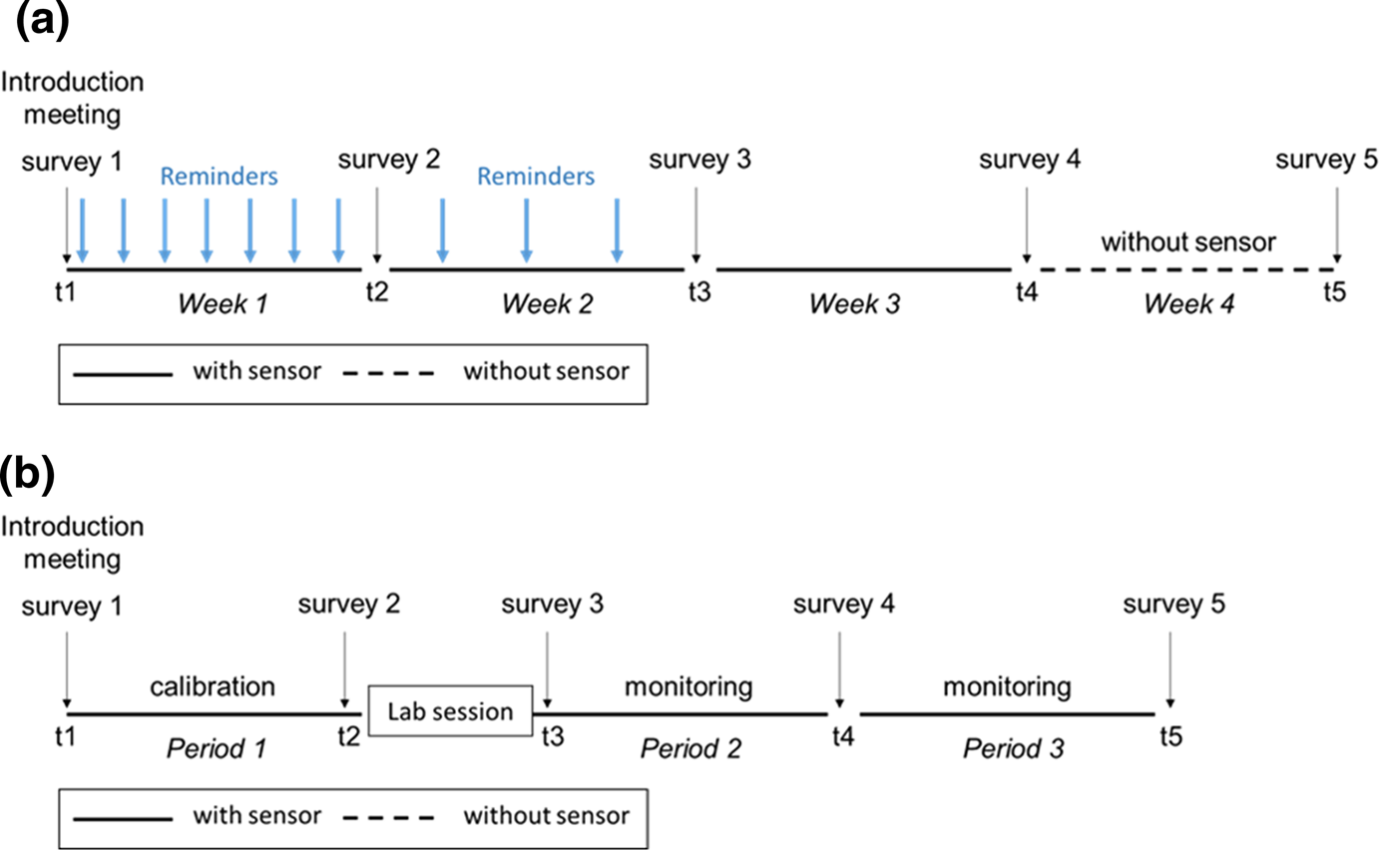
Timeline of **a** Study 1 and **b** Study 2

***Study 1*** Study 1 was a 4-week intervention program during which study participants were persuaded to change their oral health routine from brushing their teeth once a day to brushing twice a day. The main outcome variable was whether they complied with the new target brushing behavior (i.e., brushing also in the morning or in the evening) on each day during the study period. At the beginning, a face-to-face meeting was held between the experimenter and each participant. During this meeting, participants were introduced to the study and the intervention, signed a consent form, and were given a sensor to be attached to their own toothbrush. After participants returned home, their toothbrushing behaviors were monitored by the sensors for 3 weeks, and at the end of the third week they returned the sensor to the experimenter. To facilitate habit formation at the beginning, reminders for the target brushing behaviors were sent daily in the first week and every other day in the second week using a self-programmed mobile app. The reminders were cancelled after the second week since we were interested in whether the newly learned behavior could be maintained by habit alone. At the end of each week, a short survey was sent using the same app to ask questions about attitude and habit strength. (see Fig. 3a for the timeline of Study 1).

***Study 2*** Study 2 was a multi-phase intervention program during which participants were persuaded to develop an optimal oral health routine of two brushing sessions that both last for at least 2 minutes (or at least a 4-minute brushing daily). The main outcome variable was whether they brushed their teeth twice a day or not. In the beginning, participants came to the laboratory in groups of 10–15 for an introduction session, in which general study information and procedure were explained, but not the specific intervention. Also in the meeting, participants were offered new manual toothbrushes with sensors attached and were asked to sign a consent form and to complete the first survey. After the baseline period of about 5–10 days, they were invited back to the laboratory for the intervention session individually. They were shown presentations about oral healthcare and were exposed to the intervention target of brushing twice a day for at least 4 minutes. During the laboratory session, additional intervention techniques were used and physiological data from the participants were recorded for purposes unrelated to this paper (for details, see Spelt et al. 2020). The second and third survey, with mostly identical questions, was completed by the participants before and after the laboratory session. After the laboratory session, participants returned home and were monitored for a follow-up period that led to a total of approximately 3 weeks. Two additional surveys were sent by e-mail in the middle and at the end of the follow-up period. (see Fig. 3b for the timeline of Study 2)

### Participants

***Study 1*** Forty healthy university students or young graduates were recruited through a local participant database and personal network. The main inclusion criterion was that they used to only brush their teeth once a day (or at least rarely brushing twice), and the criterion was checked by personal communication with the participants. This highly educated sample consisted of 26 males and 14 females, and the average age was 24.48 (SD = 3.13, median = 24). All participants entered a lottery and eight were randomly selected to win a prize of 25 euros. The study was reviewed and approved by an ethical review board at Eindhoven University of Technology.

***Study 2*** Study 2 was conducted in collaboration with Philips Research. Seventy-nine adults from diverse educational and professional backgrounds were recruited through a recruitment agency contracted by Philips. A lenient main criterion was used that the participants used to brush only once a day, or they usually brushed less than two minutes for each session. Other criteria include that they were between 18 and 60 years old, understood Dutch, and were manual toothbrush users. The eventual sample consisted of 41 females and 37 males (one chose “other”), with ages between 20 and 63 years old (mean = 39.63, median = 38, SD = 10.97). Most participants were healthy, except that one suffered from cystic fibrosis and one from narcolepsy. Each participant was paid 80 euros by the recruitment agency. The study was reviewed and approved by the Internal Committee on Biomedical Experiments (ICBE) at Philips Research.

### Measurements

**Fig. 4:**
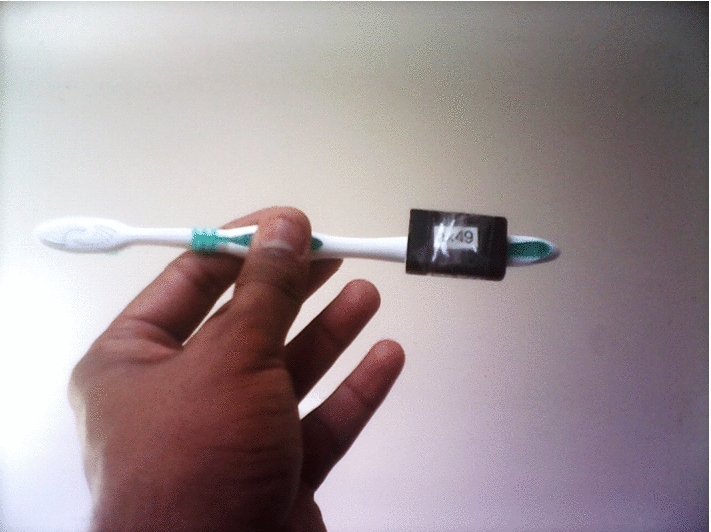
An example of how the Axivity AX3 accelerometer was attached to a toothbrush in the studies

***Toothbrushing behavior*** Participants’ toothbrushing behavior was measured by the Axivity AX3 sensors attached to the lower-end of their toothbrush grips (see Fig. 4). The Axivity AX3 sensor is a 3-axis accelerometer developed by Newcastle University specifically for scientific research on human movements (Doherty et al. 2017). Constrained by the memory space of the device, the sampling frequency was set at 50 Hz to ensure the storage of data for three weeks. The sensitivity range for accelerations was set at ±8g. The sensor was waterproof, and a fully-charged sensor could work for 3 weeks without additional charges. Participants in both studies also self-reported on how many days of the previous week they brushed their teeth in the morning/evening (Study 1) or brushed teeth twice a day for at least 2 minutes each time (Study 2).

***Habit strength*** Habit strength was measured using the 4-item Self-report Behavior Automaticity Index (SRBAI) with 7-point response scales (Gardner et al. 2012). It assessed behavioral automaticity by prompting participants to rate their agreements with descriptions of performing a target behavior (e.g., *“Behavior X is something...”*), including *“I do automatically”*, *“I do without having to consciously remember”*, *“I do without thinking”*, and *“I start doing before I realize I am doing it”*. The target behavior in Study 1 was *“brushing my teeth in the morning”* or *“brushing my teeth in the evening”*, depending on which behavior was not performed by each participant before the study. In Study 2, because of the lenient inclusion criterion, the behavior was more generally phrased as *“brushing my teeth twice a day and in total at least 4 minutes”*. Internal reliabilities of the SRBAI were very high in both Study 1 (Cronbach’s $\alpha$ = 0.95) and Study 2 (Cronbach’s $\alpha$ = 0.94). These items were translated into Dutch in Study 2.

***Attitude*** Attitude was measured using 7-point semantic differential scales that were typically used in studies that followed the Theory of Planned Behavior (Verplanken et al. 1997). Four items were used in Study 1 (*brushing my teeth in the morning/evening is:* bad–good, useless–useful, harmful–beneficial, unpleasant– pleasant), while in Study 2 three more items were added (*brushing my teeth twice every day is:* foolish–wise, unhealthy–healthy, difficult–easy). We also made a common distinction between instrumental attitude and affective attitude (Tobias 2009), because inter-item correlations and factor analysis clearly suggested that there were two separate factors. Instrumental attitude focuses on how a behavior satisfied instrumental goals, such as health benefits in the context of dental behaviors, while affective attitude taps more onto the emotional aspects of the experience relating to the behavior (e.g., comfort of brushing, effort spent on brushing). The affective attitude score was based on a single item in Study 1 (unpleasant–pleasant) and the average score of two items in Study 2 (unpleasant–pleasant, difficult–easy). Internal reliabilities (Cronbach’s $\alpha$) for instrumental attitude were 0.94 and 0.93 for the two studies, while affective attitude also had a satisfying internal reliability of 0.71 in Study 2. The attitude items were translated into Dutch in Study 2.

### Preprocessing

Preprocessing was performed to transform the raw 3-axis accelerometer data into the outcome variable to be predicted at the day-level, i.e., whether a person performed the target toothbrushing behavior or not on a specific day^4^. First, using the default Axivity AX3 software, the 3-axis signals were converted to a vector of signal vector magnitudes (SVM), which quantified the total movement magnitudes by integrating the accelerations on the x, y, and z-axis. The raw data were also down-sampled from 50 Hz to 1 Hz, so the output represented the average movement magnitude per second for the whole study period. Second, a threshold-based algorithm was used to scan the data sequentially to extract all potential brushing episodes and then a manual check was performed to exclude invalid episodes. Through this step, discrete brushing episodes were identified for each participants with timestamps, separated from rest states and non-brushing movements.

In the third step, the identified episodes were classified into 6 categories based on the starting time of the episodes: *morning* (5:00–12:00), *early afternoon* (12:00– 15:00), *late afternoon* (15:00–19:00), *early evening* (19:00–21:00), *late evening* (21:00–24:00), and *midnight* (0:00–5:00). At the data level, two variables—*morning brushing* and *evening brushing*—were created, and their values (0 or 1) were determined by searching in the relevant categories on the same date to see if any episode existed. For *morning brushing*, the category *morning* was searched for first, and if no episode was found, the category *early afternoon* was searched for. For *evening brushing*, the categories *late evening* and *midnigh*t were searched for first, and if no episode was found, the category *early evening* was searched for. Brushing episodes that were not counted as morning or evening brushing (e.g., brushing one’s teeth in the middle of the day) were disregarded because they were unrelated to the context-specific brushing behaviors. When there were known or unknown events that caused noise in the data in a certain period, the values for the two brushing variables were coded as missing data. Finally, at the day level, dichotomous indicators (0 or 1) for *the target brushing behavior* and for *brushing twice* were used as the outcome variable in Study 1 and Study 2 respectively. After the last step, four participants from Study 1 and three participants from Study 2 were removed from further analyses due to their large pecentage of missing sensor data.

### Model comparison

The target for prediction was the brushing behavior on the next day, with the occurrence of brushing as the *negative cases* and the absence of brushing as the *positive cases*. They were coded in this way because for real applications a potentially more important goal would be to detect the positive cases, i.e., the days on which the brushing behavior was likely to be omitted. To compare the theory-based approach with the survey approach and the past-behavior approach, logistic regression models with 5 different feature sets were compared^5^:

- *Survey model*: The primary features in the survey model were the variables measured by weekly surveys, including *instrumental attitude*, *affective attitude*, and *self-reported behavioral automaticity*. In addition, the *occurrence of laboratory sessions* (including the introduction meeting in Study 1) and the *occurrence of reminders* (including notifications and e-mails for surveys) were also included as features.
- *Past-BR model*: The primary feature in this model was the past behavior rate (BR) until the day of the last observation. For example, if the brushing behavior on the $11^{th}$ day was to be predicted, the brushing rate in the last 10 days (e.g., 0.8) would be the value for this variable. For the first day, self-reported behavior rates in the previous week were used for the initial values. Again, the *occurrence of laboratory sessions* and the *occurrence of reminders* were also included as features.
- *Past-BR7 model*: A variant of the past-BR model was to use the behavior rate of the last 7 days, thus focusing only on recent behavioral information. In case of time points with less than 7 days in the past, behavior rate since the start was used (i.e., the same with past-BR model).
- *Weighted past-BR model*: Another way to focus more on recent data was to add a temporal discounting parameter when counting past behavior frequency. This weighted behavior rate feature used to predict behavior at time *t* was computed as $\sum _{i=1}^{t-1} B_i\times \gamma ^{(t-i)}/(t-1)$, with *B* as the behavior indicator at time *i* (0 or 1). The optimal value for parameter $\gamma$ was found through the two-level hierarchical cross-validation procedure described below.
- *Theory-based model*: This was the model of our interest that includes only computed *habit strength* and *accessibility* as features.

**Fig. 5:**
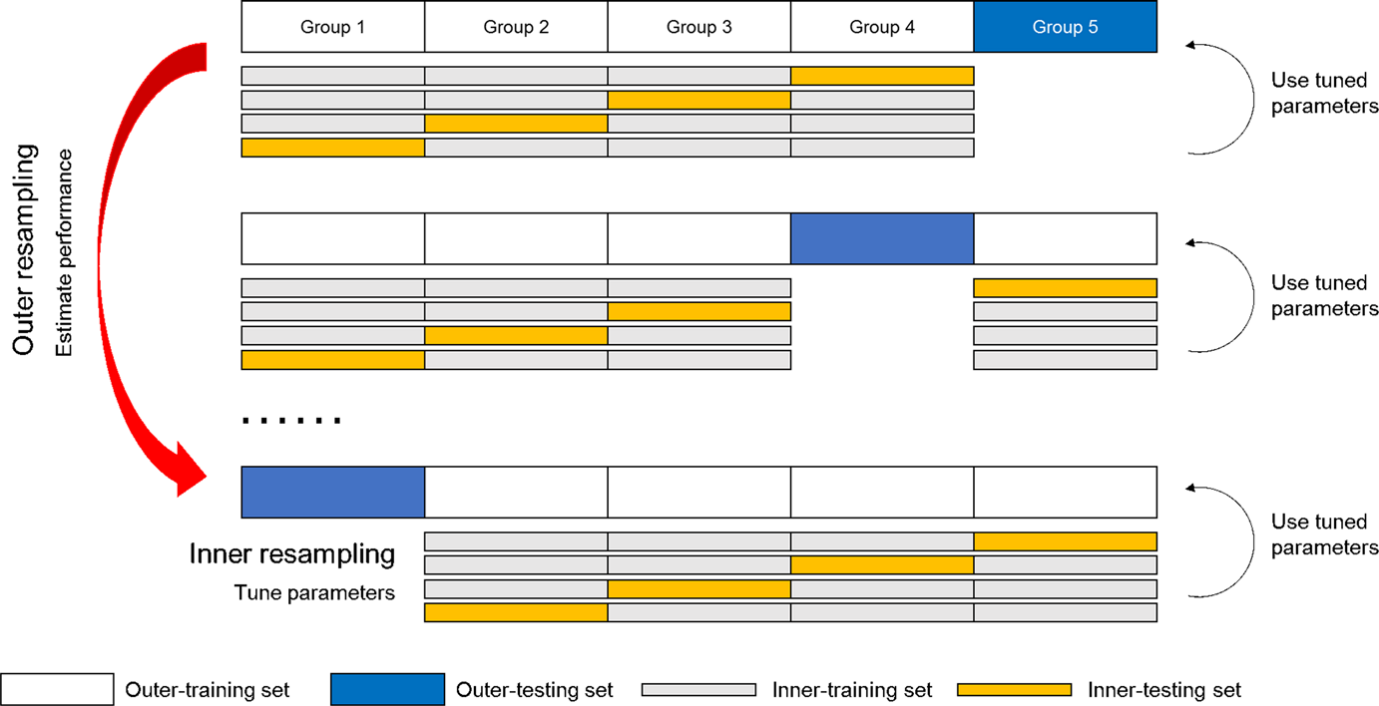
An illustration of the nested cross-validation procedure used (it shows the 5-fold scenario for Study 2, but the same idea applies to Study 1)

Two different approaches were used to compare model performance. First, a two-level hierarchical *k*-fold cross-validation procedure was used on each of the two data sets separately (see Fig. 5). For each data set, all observations were divided into *k* non-overlapping groups (with the restriction that one participant’s data were always in only one group), so that 1 group was reserved for model testing, and the remaining *k*-1 groups were used for training in each round (the outer loop). Because tuning was needed for the free parameters in the equations for habit strength, memory accessibility, and weighted past behavior rate, the training set in each round was further divided, with 1 group reserved as the testing set for parameter tuning and the remaining *k*-2 groups as the training set for parameter tuning (the inner loop). For each free parameter in the theory-based model, a 1000-step random search was used, and in each step a random value was drawn from a uniform distribution between 0 and 1. For the discounting parameter $\gamma$, a 100-step grid search was used, including values between 0.01 and 1 with a step size of 0.01. These parameter values were optimized to obtain the best overall prediction performance in the inner cross-validation loop, indicated by area under curve (AUC) in receiver operating characteristic (ROC) curves. Due to the sample size difference between the two studies, 9- fold was used for Study 1 (4 participants in each group) and 5- fold was used for Study 2 (15 participants in each group), in order to have sufficient data for training.

Since we had two similar data sets, in a second approach, we evaluated the ability of each model type to predict new data. Specifically, one of the two data sets was used to train the models, and the resultant models were used to predict the observations in the other data set. When parameter tuning was required, a *k*-fold cross-validation was used on the whole training data set, with the same search methods indicated above. Again, 9-fold or 5-fold cross-validation was used when Study 1 or Study 2 was used as the training data set respectively. This approach was used mainly to evaluate the generalizability of the parameters used to compute theory-based features (e.g., HGP, ADP) across intervention trials.

For model comparison, we primarily focused on AUC. Compared with other performance metrics, AUC takes both positive and negative cases into account and is generally considered the best for both balanced and unbalanced data sets (Halimu et al. 2019). AUC was also chosen because we were more interested in predicted probabilities of brushing rather than the classifications under a particular threshold. As recommended by previous research (Dietterich 1998; Raschka 2018), we also used the McNemar’s Chi-squared test to test whether the prediction performance differences between pairs of models were statistically significant. This test basically examines whether the correct and incorrect predictions would match the expected distribution under the assumption that two models are equally good. In addition, various performance measures computed using the optimal threshold for each model, namely Matthew correlation coefficient (MCC), overall accuracy, *F*-score, true positive rate, false positive rate, precision, and negative prediction value, were also computed. All analyses were performed in R statistical programming environment (version 3.3.3), with the help of the *mlr* (machine-learning R, version 2.1.3) package (Bischl et al. 2016).

## Results

### Performance within individual datasets

**Fig. 6:**
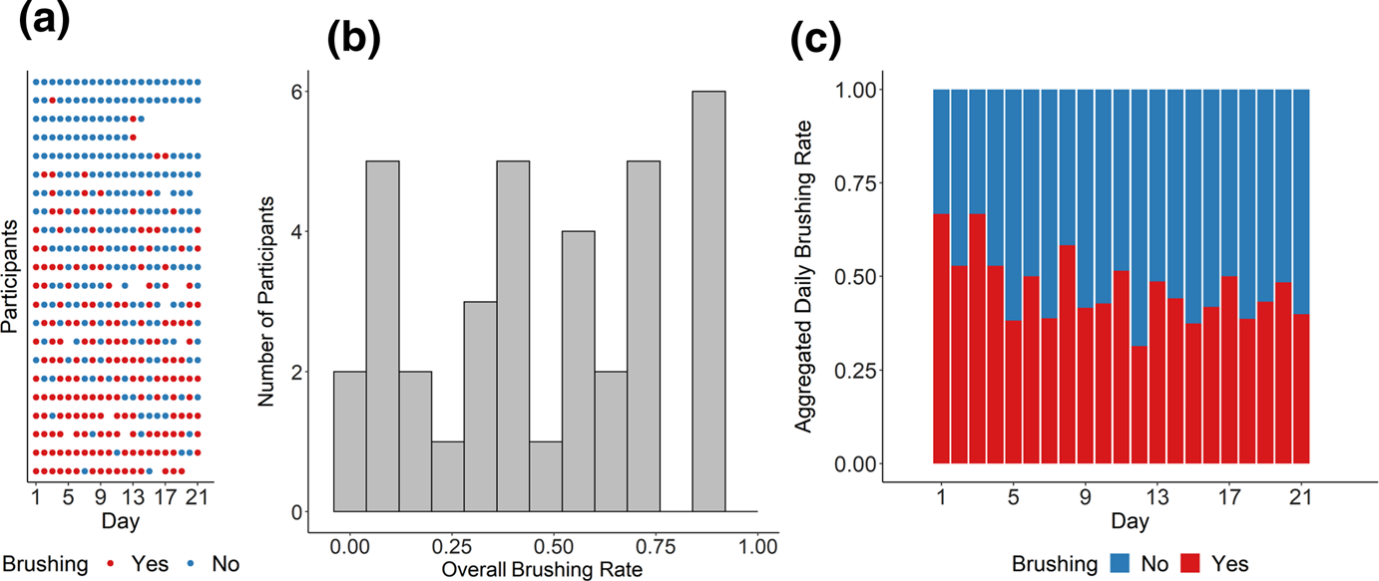
Descriptives of Study 1. **a** Raw brushing data of each participant over the course of Study 2. Each row represents a participant (ordered from top to bottom based on brushing frequencies from low to high) and each column represents a day. **b** Histogram of individual participants’ brushing rates over the course of the study. **c** Change of daily brushing rate over time (aggregated over all participants)

**Fig. 7:**
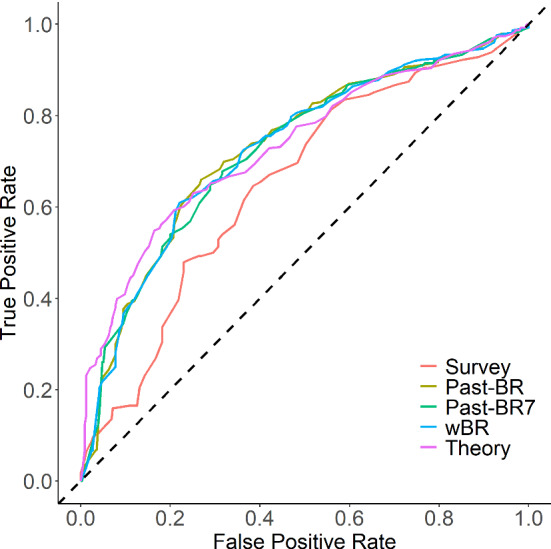
Model comparison results of Study 1 based on ROC curves for different models (*Note:* wBR, weighted past-BR model; Theory, theory-based model)

***Study 1*** Study 1 included 711 non-missing observations from 36 participants for the prediction task, with 376 positive cases (non-brushing) and 335 negative cases (brushing) (for more detailed descriptives, see Fig. 6). Thus, the prediction accuracy would be 53% if a no-skill model always predicts positive cases. Figure 7 shows the testing ROC curves of different models, and Table 2 compares additional testing performance measures of the models (aggregated over cross-validation iterations)^6^. All models were able to perform substantially better than the no-skill model, with average accuracy ranging between 63.2% and 69.3%. McNemar’s Chi-squared tests suggested that the theory-based model performed clearly better than the survey models ($\chi ^2$(1) = 6.48, *p* = .011) and just as good as the three models using different summaries of past behavior as features (all *p*s $> .701$). Parameter values optimized for the theory-based model were 0.10 for HDP, 0.20 for HGP, 0.28 for ADP, 0.13 for $\text {AGP}_\mathrm{beh}$, and 0.26 for $\text {AGP}_\mathrm{rem}$, averaged over the 9 repetitions. For the weighted past-behavior model, the optimized parameter $\gamma$ was 0.98 on average.

**Table 1:** Comparison of model performances in predicting testing data (Study 1)

	AUC	MCC	Acc	TPR	FPR	Precision	F1-score	NPV
Survey	0.652	0.261	0.632	0.646	0.385	0.653	0.650	0.608
Past-BR	0.730	0.391	0.693	0.660	0.269	0.734	0.695	0.657
Past-BR7	0.727	0.361	0.681	0.678	0.316	0.706	0.692	0.654
wBR	0.727	0.392	0.689	0.609	0.221	0.756	0.675	0.640
Theory	0.734	0.390	0.686	0.593	0.209	0.761	0.667	0.634

wBR, weighted past-BR model; Theory, theory-based model; Acc, accuracy; TPR, true positive rate; FPR, false positive rate; NPV, negative prediction value; MMC, Matthews correlation coefficient

**Fig. 8:**
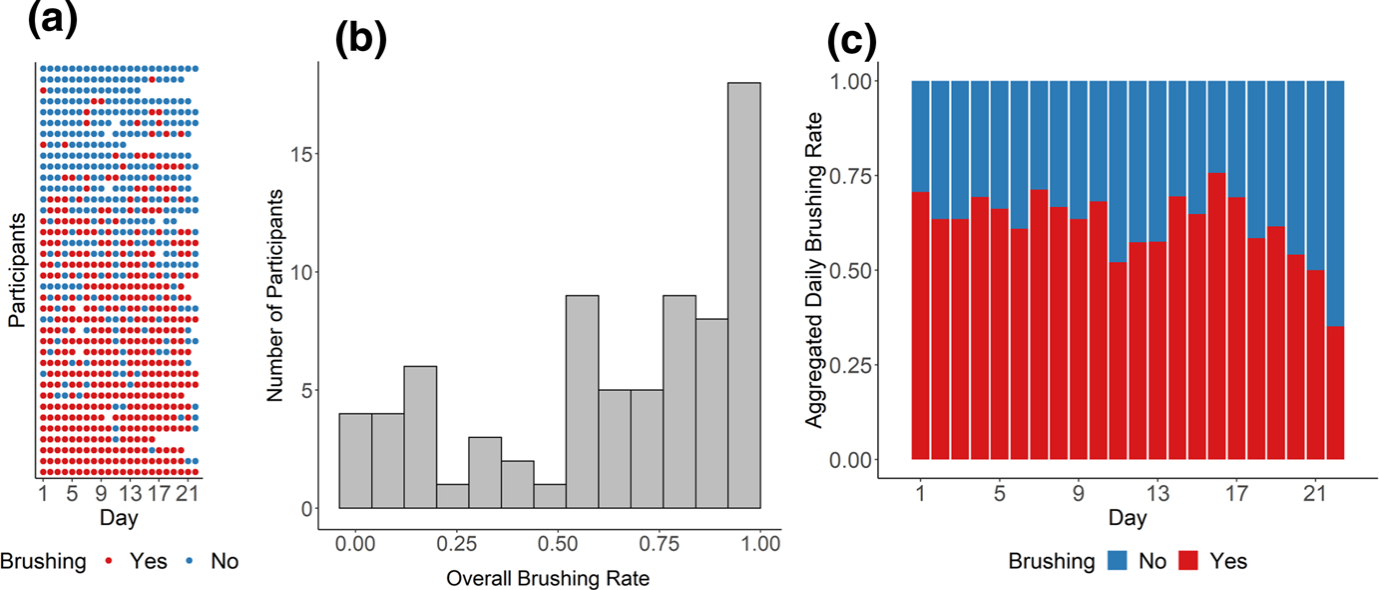
Descriptives of Study 2. **a** Raw brushing data of each participant over the course of Study 2. Each row represents a participant (ordered from top to bottom based on brushing frequencies from low to high) and each column represents a day. **b** Histogram of individual participants’ brushing rates over the course of the study. **c** Change of daily brushing rate over time (aggregated over all participants)

***Study 2*** Study 2 included 1508 non-missing observations from 75 participants for the prediction task, with 557 positive cases (non-brushing) and 951 negative cases (brushing) (for more detailed descriptives, see Fig. 7). Thus, the data were less balanced and the prediction accuracy would be 63% if a no-skill model always predicts negative cases. Figure 8 shows the testing ROC curves of different models, and Table 3 compares additional testing performance measures of the models in Study 2^7^. Since the data were more unbalanced (more negative cases due to a higher brushing rate) compared to Study 1, all models were able to predict more accurately, with average accuracy between 66.1% and 77.6%. Like in Study 1, McNemar’s Chi-squared tests showed again that the theory-based model performed much better than the survey models ($\chi ^2$(1) = 50.00, *p* < .001) and was on par with the three models based on past behavior (all *p*s > .124). Parameter values optimized for the theory-based model were 0.19 for HDP, 0.30 for HGP, 0.64 for *ADP*, 0.58 for AGP$_\mathrm{beh}$, and 0.27 for AGP$_\mathrm{rem}$, averaged over the nine repetitions. For the weighted past-behavior model, the optimized parameter $\gamma$ was 0.97 on average.

**Fig. 9:**
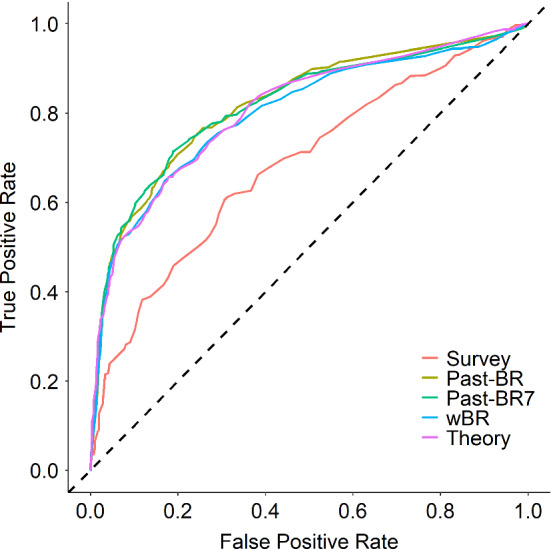
Model comparison results of Study 2 based on ROC curves for different models (*Note:* wBR, weighted past-BR model; Theory, theory-based model)

**Table 2:** Comparison of model performances in predicting testing data (Study 2)

	AUC	MCC	Acc	TPR	FPR	Precision	F1-score	NPV
Survey	0.684	0.293	0.661	0.605	0.306	0.537	0.569	0.750
Past-BR	0.819	0.495	0.752	0.767	0.257	0.636	0.695	0.845
Past-BR7	0.820	0.523	0.776	0.715	0.188	0.690	0.702	0.829
wBR	0.800	0.486	0.763	0.648	0.169	0.692	0.669	0.801
Theory	0.809	0.482	0.761	0.657	0.179	0.683	0.670	0.803

wBR, weighted past-BR model; Theory, theory-based model; Acc, accuracy; TPR, true positive rate; FPR, false positive rate; NPV, negative prediction value; MMC, Matthews correlation coefficient

### Performance across the two datasets

**Fig. 10:**
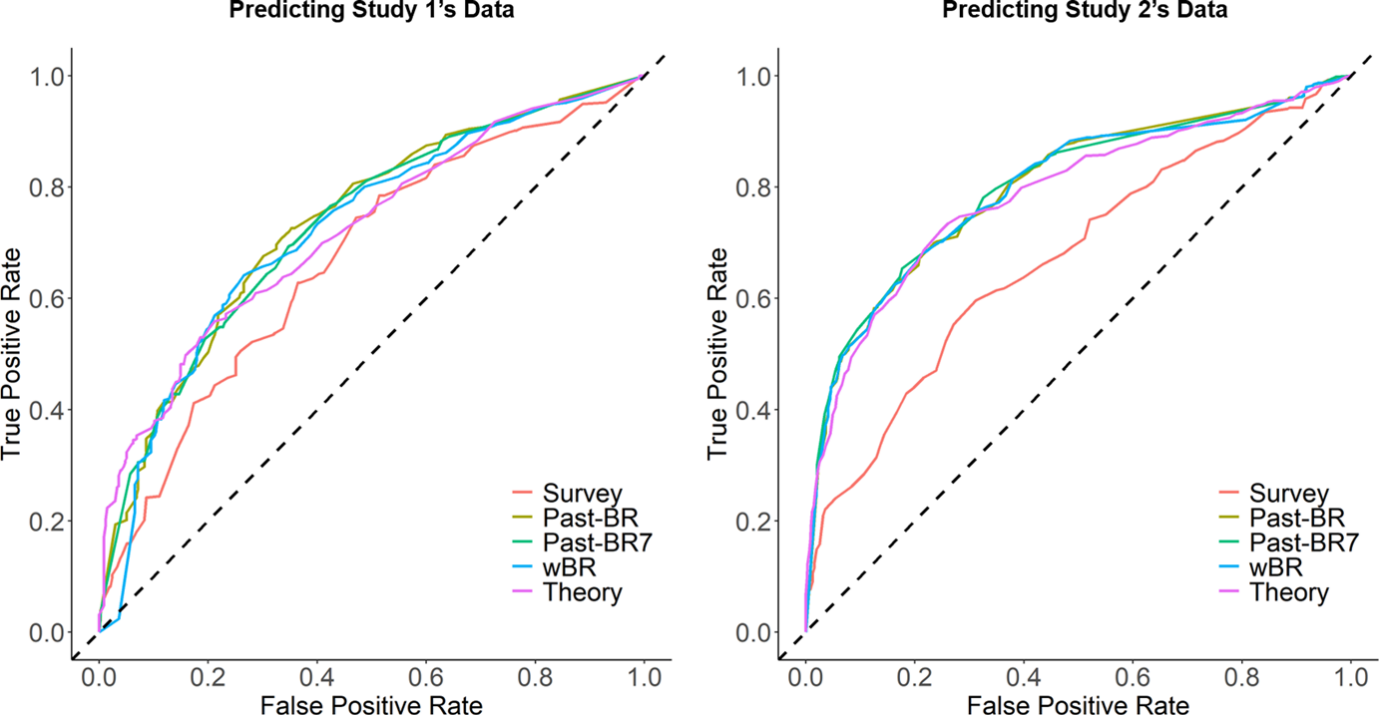
Model comparison results in terms of predicting new data, based on ROC curves of different models (*Note:* wBR, weighted past-BR model; Theory, theory-based model)

The results of the models’ abilities for predicting unseen data from a different study are summarized in Fig. 10 and Table 3,^8^. Overall, the theory-based model outperformed the survey model when predicting Study 2’s data ($\chi ^2$(1) = 39.91, *p* < .001) but not when predicting Study 1’s data ($\chi ^2$(1) = 1.08, *p* = .299). There were again no reliable differences between the theory-based model and the models based on past behavior (Study 1: all *p*s > .211; Study 2: all *p*s > .065). When comparing the cross-dataset results and the within-dataset results (Sect. 5.1), there was a general trend that predicting new data led to slightly worse performance (but except for the survey, Past-BR, and Past-BR7 models when predicting Study 1’s data), but all the differences were not statistically significant (Study 1: all *p*s > .462; Study 2: all *p*s > .241).

**Table 3:** Comparison of model performances in predicting new data

		AUC	MCC	Acc	TPR	FPR	Precision	F1-score	NPV
Predicting dataset 2	Survey	0.669	0.280	0.643	0.745	0.472	0.639	0.688	0.648
	Past-BR	0.740	0.373	0.686	0.676	0.301	0.715	0.695	0.657
	Past-BR7	0.736	0.345	0.674	0.691	0.346	0.691	0.691	0.654
	wBR	0.722	0.376	0.685	0.641	0.266	0.730	0.683	0.646
	Theory	0.719	0.354	0.667	0.559	0.212	0.747	0.639	0.614
Predicting dataset 1	Survey	0.672	0.278	0.654	0.596	0.312	0.528	0.560	0.744
	Past-BR	0.789	0.456	0.741	0.700	0.236	0.635	0.666	0.813
	Past-BR7	0.763	0.482	0.761	0.654	0.177	0.684	0.669	0.802
	wBR	0.796	0.463	0.747	0.680	0.213	0.651	0.665	0.808
	Theory	0.788	0.463	0.741	0.720	0.246	0.631	0.673	0.821

wBR, weighted past-BR model; Theory, theory-based model; Acc, accuracy; TPR, true positive rate; FPR, false positive rate; NPV, negative prediction value; MMC, Matthews correlation coefficient

### Parameter estimation

Lastly, for theoretical interests, we examined the optimal parameter values for the free parameters in the theory-based equations of habit strength and accessibility. For parameters governing the dynamics of habit strength, optimal ranges of parameter values could be found, and the results were similar regardless of the data set used (see Fig. 10). To achieve best performance based on AUC, the optimal value for the habit decay parameter (HDP) was in the range of 0.15 and 0.2, while the optimal value for the habit gain parameter (HGP) was in the range of 0.1 and 0.3.

**Fig. 11:**
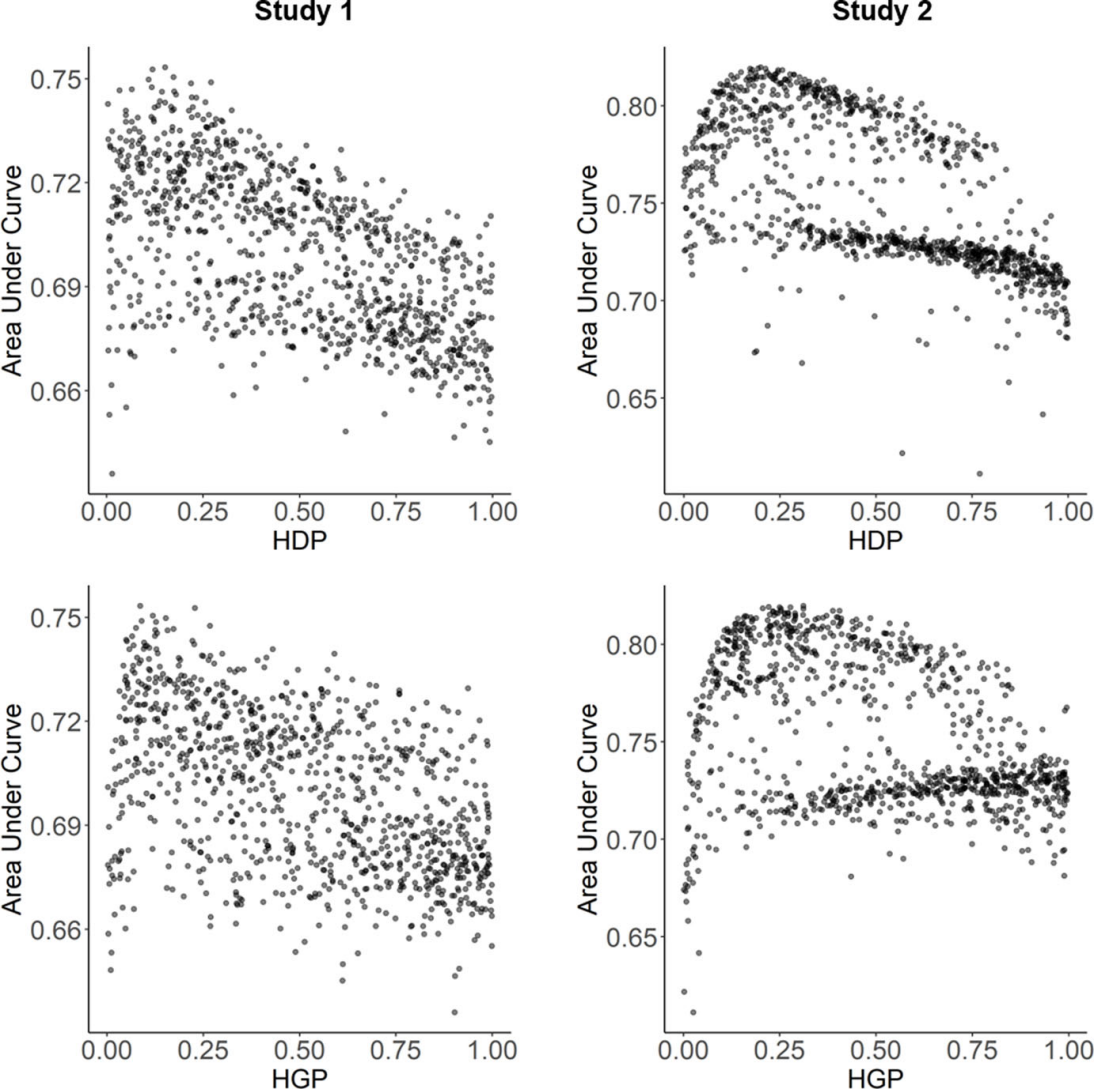
Tuning results for parameter HDP and HGP in the computational model of habit strength, shown as the relationship between parameter values (x-axis) and model performance (area under curve, y-axis)

In contrast, for parameters that determine the dynamics of accessibility, there was no clear relationships between their values and model prediction performance (see Fig. 11). If one examined the importance of individual features in the theory-based models, habit strength was 2.46 times and 4.71 times more important than memory accessibility in Study 1 and Study 2, respectively.

**Fig. 12:**
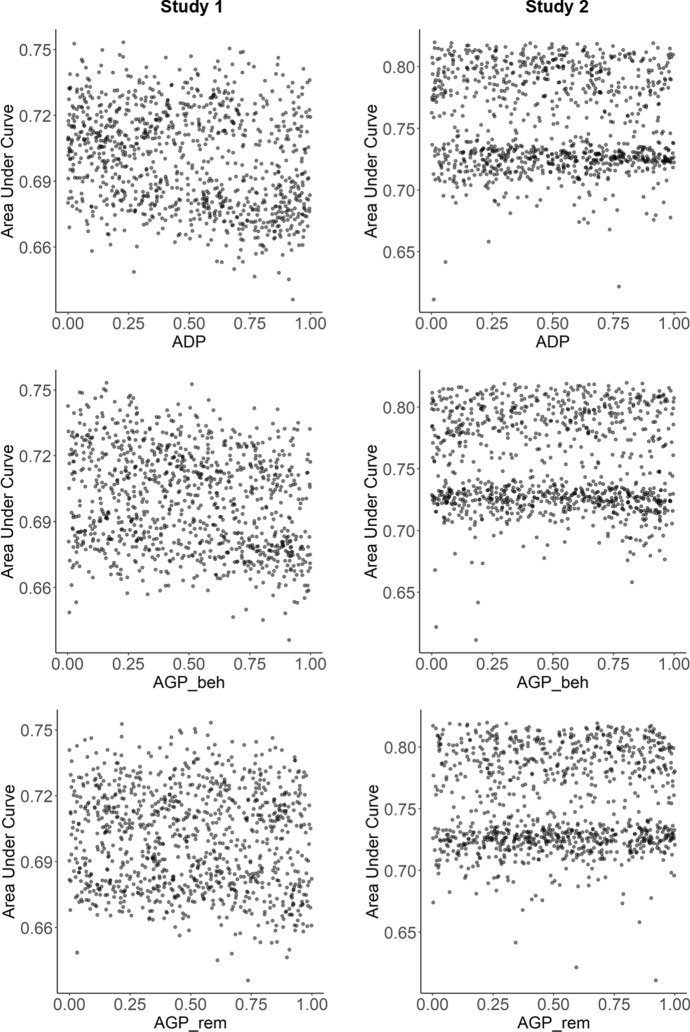
Tuning results for parameter *ADP*, $AGP_{beh}$, and $AGP_{rem}$ in the computational model of memory accessibility, shown as the relationship between parameter values (x-axis) and model performance (area under curve, y-axis)

## General discussion

### Summary of results

Recently developed theory-based computational models allow BCSSs to model users’ habit learning in behavior change processes. In this paper, we reviewed the computational models of habit learning and evaluated whether computing habit strength could improve behavior prediction, based on data collected in two field intervention studies on toothbrushing behavior. Through a nested cross-validation procedure, a theory-based model that computed habit strength and memory accessibility were compared with four baseline models, in terms of how well they could predict brushing behavior on the next day. In both studies, the theory-based model performed better than the survey model that used self-reported behavioral determinants as features, but its performance was only as good as the three models that relied on theory-free summaries of past behavior. A similar pattern was found when we used models trained from one dataset to predict the cases in the other dataset. The theory-based approach showed reasonable generalizability across the two intervention trials since prediction performance did not drop significantly for new data without re-optimizing the free parameters.

### Implications for BCSS and habit Research

While these results do not support an unique advantage of the theory-based approach, they provide very useful information regarding the important task of behavior prediction for BCSS. For the sole purpose of behavior prediction, tracking past behavior and summarizing it in a sensible way might be sufficient without bothering the users to self-report their motivation, attitude or habit strength. While the equation of habit strength was motivated by theories (Klein et al. 2011; Miller et al. 2019), the computed variable can be considered as a specific summary of past behavior as well. In fact, similar to the weighted past-BR model, which discounts distant behaviors, the equation of habit strength also discounts the contributions of behaviors that are far in the past in an exponential way, given by the decay parameter to the power of n (HDP$^n$), where n denotes the number of time steps in the past. But unlike the weighted past-BR model, behaviors in the later stage of habit formation also tend to have increasingly smaller contributions to overall behavior summary because the habit gain parameter is modulated by the term 1 - $HS_t$. Despite these properties, our data suggest that the two sophisticated summaries of past behavior are not superior to a simple calculation of past behavior rate. Given its simplicity and robustness (i.e., no need for parameter tuning), simple past behavior rate should be preferred as a feature for predictive modeling in behavior change interventions.

For real-world applications, BCSSs can simply estimate the probability of brushing (non-brushing) and then use different thresholds for delivering different types of interventions. For example, if brushing probabilities stay very low for several days (e.g., 10%), the system may decide to repeat an extensive education session about the importance of an optimal oral health routine. Instead, if a user is predicted to brush the next morning with a probability of 0.6, a gentle reminder may be sent. Such adaptive interventions are important because even though the costs of delivering digital interventions are low, too frequent or inappropriate actions may disrupt or even irritate users (Mehrotra et al. 2016). Besides behavior prediction, a system may use the computed habit strength more directly. For example, tracking a user’ habit strength of a newly adopted behavior may give the system a better idea about the progress of behavior change. Even when the target behavior is already performed consistently, a habit strength weaker than a certain threshold (e.g., 0.8) would suggest that the current intervention should be continued to reduce the risk of relapse.

Besides the implications for behavior prediction and intervention, the parameter estimation procedure used in our studies also has implications for the theoretical understanding of habit formation. The optimal values tuned for the habit gain parameter are very close to the corresponding values of 0.19 obtained through a statistical modeling of the temporal dynamics of self-reported habit strength or behavioral automaticity (Lally et al. 2010). However, inconsistent with previous studies that suggested much smaller habit decay parameter (Tobias 2009; Lally et al. 2010), its value was in the same range with the habit gain parameter in our studies. In general, these results speak to the theoretical meaningfulness of the computational model of habit strength used for prediction. In contrast, the parameters in the equation of accessibility did not seem to have optimal values, which casts doubts onto the validity of modeling memory accessibility in its current form.

### Limitations and future work

First, our research was limited by the types of data we could collect during the two intervention studies. Because habits are theorized as context-dependent, the presence or absence of behavior-associated contexts or cues needs to be monitored. Instead, we assumed that the participants were always brushing their teeth in the same environments and thus $Cue_{t}$ for computing habit strength was fixed at 1. Future studies can benefit from tracking participants’ location, for example, whether they are at home in the evenings, in order to compute habit strength more accurately. Moreover, toothbrushing behavior or any lifestyle behavior in daily life is also influenced by the immediate internal and external states of a person. For example, when someone is very tired in an evening, they are more likely to skip toothbrushing and go directly to bed. On the contrary, the presence or absence of one’s family member at night may change the social pressure to comply with an optimal dental routine. Measuring these context factors in future studies may further improve the prediction accuracy of the current best models (i.e., 70–78%).

Second, while there are several computational models of habit formation in the literature (Miller et al. 2019; Psarra 2016; Tobias 2009), we focused on testing the model by Klein and colleagues (2011). In future work, researchers may want to compute habit strength using the different models from the literature and more systematically compare their contributions to behavior prediction. However, given the relative small differences between the models and the measurement errors usually introduced in real-world intervention studies, we doubt that using a different model would drastically change the answers to the main research questions.

One final limitation is the inclusion of only toothbrushing behavior in our evaluation studies. It is reasonable to question whether our findings can be generalized to other behavioral domains, such as physical activities or dietary behaviors. Despite this limitation, we wish to highlight that while different behaviors are regulated through different processes, the mechanism that link behavior repetition to habit formation is the same in theory. Hence, the computational models of habit formation are supposed to be domain-general models and our idea of computing habit strength for behavior prediction should also be widely applicable. Still, as different habits may change faster or slower and they may influence actual behavior to a greater or lesser extent, parameter estimations and prediction performances can vary across domains (Stawarz et al. 2015). We hope our work will stimulate more interest in combining theory-based computational modeling and data-driven methods for behavior prediction and intervention in various application domains of BCSSs.

## Footnotes

1 Tobias’s equation does not simulate habit decay while in isolation, but one should note that the equation for habit formation is meant to be used together with other equations in a complex model of behavior change.

2 In a more recent unpublished work, we also found that Klein’s model fitted the empirical data of forming a habit of hand washing in the COVID-19 context better than the alternative models.

3 Data, analysis script, and other materials can be found in a Open Science Framework repository: https://osf.io/adkb4/.

4 A more technical description of the preprocessing steps can be found in Zhang (2019) and the code is accessible on Open Science Framework: https://osf.io/adkb4/. A similar procedure of detecting toothbrushing episodes was validated against self-reports in an internal study at Philips Research.

5 We tried other machine learning algorithms such as support vector machine and random forests, but they performed equal to or slightly worse than the simple logistic regression. Since different algorithms did not affect the model comparison results, we only report the logistic regression results. We also found that combining features from the different models (e.g., theory-based model and past-BR model) did not lead to better prediction performance, so the detailed results are not reported.

6 Thresholds used for the logistic regression models were 0.5 (survey), 0.55 (past-BR), 0.51 (past-BR7), 0.59 (weighted past-BR), and 0.55 (theory-based).

7 Thresholds used for the logistic regression models were 0.38 (survey), 0.29 (past-BR), 0.36 (past-BR7), 0.41 (weighted past-BR), and 0.36 (theory-based).

8 When predicting Study 1’s data, thresholds used for the logistic regression models were 0.31 (survey), 0.33 (past-BR), 0.32 (past-BR7), 0.37 (weighted past-BR), and 0.37 (theory-based). When predicting Study 2’s data, thresholds used for the logistic regression models were 0.57 (survey), 0.53 (past-BR), 0.57 (past-BR7), 0.56 (weighted past-BR), and 0.57 (theory-based).
